# Absence of knockdown mutations in pyrethroid and DDT resistant populations of the main malaria vectors in Colombia

**DOI:** 10.1186/s12936-019-3034-1

**Published:** 2019-12-02

**Authors:** Lorena I. Orjuela, Diego A. Álvarez-Diaz, Juliana A. Morales, Nelson Grisales, Martha L. Ahumada, Juan Venegas H, Martha L. Quiñones, María F. Yasnot

**Affiliations:** 1 Grupo de Investigaciones Microbiológicas y Biomédicas de Córdoba-GIMBIC, Universidad de Córdoba, Montería, 230001 Colombia; 2 Universidad de Cartagena, Facultad de Medicina, Sede Zaragocilla, Calle 30 N° 48-152, Cartagena de Indias, Bolívar 1300 Colombia; 3 Grupo de Salud Materna y Perinatal, Dirección de Investigación en Salud Pública, Instituto Nacional de Salud, Bogotá D.C., 110111 Colombia; 4 Grupo de Entomología, Dirección de Investigación en Salud Pública, Instituto Nacional de Salud, Bogotá D.C., 110111 Colombia; 5 Zika AIRS Project, Abt Associates, Rockville, MD 20852 USA; 6 Programa de Biología Celular y Molecular, Instituto de Ciencias Biomédicas (ICBM), Facultad de Medicina, Universidad de Chile, Santiago de Chile, 8320000 Chile; 7 Departamento de Salud Pública, Universidad Nacional, Bogotá D.C., 110111 Colombia

**Keywords:** *Anopheles albimanus*, *An. darlingi*, *An. nuneztovari* s.l., kdr, Insecticide resistance

## Abstract

**Background:**

Knockdown resistance (kdr) is a well-characterized target-site insecticide resistance mechanism that is associated with DDT and pyrethroid resistance. Even though insecticide resistance to pyrethroids and DDT have been reported in *Anopheles albimanus*, *Anopheles benarrochi* sensu lato (s.l.), *Anopheles darlingi*, *Anopheles nuneztovari* s.l., and *Anopheles pseudopunctipennis* s.l. malaria vectors in Latin America, there is a knowledge gap on the role that kdr resistance mechanisms play in this resistance. The aim of this study was to establish the role that kdr mechanisms play in pyrethroid and DDT resistance in the main malaria vectors in Colombia, in addition to previously reported metabolic resistance mechanisms, such as mixed function oxidases (MFO) and nonspecific esterases (NSE) enzyme families.

**Methods:**

Surviving (n = 62) and dead (n = 67) *An. nuneztovari* s.l., *An. darlingi* and *An. albimanus* mosquitoes exposed to diagnostic concentrations of DDT and pyrethroid insecticides were used to amplify and sequence a ~ 225 bp fragment of the voltage-gated sodium channels (VGSC*)* gene. This fragment spanning codons 1010, 1013 and 1014 at the S6 segment of domain II to identify point mutations, which have been associated with insecticide resistance in different species of *Anopheles* malaria vectors.

**Results:**

No *kdr* mutations were detected in the coding sequence of this fragment in 129 samples, 62 surviving mosquitoes and 67 dead mosquitoes, of *An. darlingi*, *An. nuneztovari* s.l. and *An. albimanus.*

**Conclusion:**

Mutations in the VGSC gene, most frequently reported in other species of the genus *Anopheles* resistant to pyrethroid and DDT, are not associated with the low-intensity resistance detected to these insecticides in some populations of the main malaria vectors in Colombia. These results suggest that metabolic resistance mechanisms previously reported in these populations might be responsible for the resistance observed.

## Background

Insecticide resistance in major malaria vectors worldwide threatens prevention and control efforts of the disease. According to the World Malaria Report 2018, resistance to the four insecticide classes available for mosquito control—pyrethroids, organochlorines, carbamates and organophosphates—is outspread in all major malaria vectors across the world [1]. Sixty-eight countries reported resistance to at least one of the four insecticide classes in one malaria vector from one collection site, and 57 countries reported resistance to two or more insecticide classes. Importantly, up to 54 countries have reported resistance to pyrethroids, the only insecticide class currently recommended by the World Health Organization (WHO) for using on long-lasting insecticidal nets (LLINs), in at least one malaria vector [1].

In Latin America, compared with the other regions of the world, studies on the evaluation of insecticide resistance are scarce and most of the reports show a susceptibility status of the main malaria vectors to the tested insecticides. *Anopheles albimanus* is the species with highest number of reports of insecticide resistance. This species has been found resistant to deltamethrin, lambda-cyhalothrin, DDT, and malathion in Colombia [2], also in Guatemala this species has shown to be resistant to deltamethrin and fenitrothion [3], in Mexico to deltamethrin, DDT and pirimiphos methyl [4], in Panama to deltamethrin, lambda-cyhalothrin, cyfluthrine and cypermethrin [5], and in Peru to deltamethrin, fenitrothion, permethrin, DDT, bendiocarb, cyfluthrine, cypermethrin and malathion [6, 7]. Other reports of insecticide resistance have been made for *Anopheles bennarochi* sensu lato (s.l.) and *Anopheles pseudopunctipennis* s.l. in Peru [6], *Anopheles darlingi* and *Anopheles nuneztovari* s.l. in Colombia [8–12], and *Anopheles aquasalis* in Venezuela [13]. According to the global report on insecticide resistance in malaria vectors 2010–2016 of the WHO [14], in Latin America, during this period, pyrethroid resistance was detected in all, but two countries, Guatemala and Nicaragua, in which monitoring was undertaken and no DDT resistance was detected at most sites tested except in Colombia, where DDT resistance has been reported since 1980 [10]. Furthermore, there was evidence of emerging resistance to carbamates, particularly in Bolivia, Ecuador and Nicaragua, and organophosphate resistance in four countries [14].

Knockdown resistance (*kdr*) is a well-characterized target-site insecticide resistance mechanism that is associated with DDT and pyrethroid resistance. *Kdr* point mutations occur in the voltage-gated sodium channel (VGSC), usually located in the transmembrane segment IIS6 or in the linker regions connecting domain III and domain IV in species of the genus *Anopheles* [15]. To date, seven mutational variations, V1010L, N1013S, L1014F, L1014S, L1014C, L1014W and N1575Y, have been reported in 13 *Anopheles* species, most of them belonging to Africa and Asia, with L1014F and L1014S being the most frequently found [16]. Recently, two additional mutations, 1048N and S1156G, in *Anopheles coluzzii* have been reported, although yet to be linked with resistance [17]. In malaria vectors of Latin America, this mechanism has been investigated in *An. albimanus, An. darlingi, An. vestitipennis* and *An. pseudopunctipennis,* and only two mutational variations, L1014F and L1014C, were identified in samples of *An. albimanus* from Mexico, Costa Rica and Nicaragua [18, 19].

In Colombia, resistance to pyrethroids, organophosphates and DDT have been reported for *An. nuneztovari* s.l. and *An. albimanus*, and in the case of *An. darlingi* also to carbamates [8–12, 20, 21]. Insecticide resistance mechanisms described are: increased metabolism through mixed function oxidases (MFO) and non-specific esterases (NSE) involved in cross-resistance between lambda-cyhalothrin and DDT in *An. darlingi* [12], and increased levels of MFO and modified acetylcholinesterase (MACE) involved with the resistance to pyrethroids and the organophosphate malathion in *An. nuneztovari* s.l. [11]. Up until now, the possibility that the *kdr*-type resistance mechanism occurs in populations where there is simultaneous resistance to DDT and to pyrethroids has not been ruled out.

Knowledge on the molecular mechanisms associated with insecticide resistance is necessary for designing appropriate vector control measures. This information allows the identification of the most effective insecticide for use by vector control programmes and predict how mosquitoes may react to the insecticides that will be used in the vector control programmes. Additionally, this knowledge can establish a baseline to assess the impact of resistance on vector control and elaborate strategies to manage it. As different resistance mechanisms show different potential to cause control failure [22], it is important to employ tests that allow the determination of the underlying genetic mechanisms responsible for the observed resistance in a surveillance monitoring programme.

In Colombia, there is a knowledge gap about the insecticide resistance mechanisms on malaria vectors. Furthermore, *kdr* resistance mechanisms, widely described for other major malaria vectors elsewhere, remain unidentified in the country. This study aims to explore the role that *kdr* mechanisms play in pyrethroid and DDT resistance in the main malaria vectors in Colombia in addition to metabolic mechanisms of resistance, such as MFO and NSE enzyme families reported previously.

## Methods

### Mosquito populations

Specimens of *An. nuneztovari* s.l., *An. darlingi* and *An. albimanus* from six localities of Valle del Cauca, Chocó and Norte de Santander Departments were selected (Table 1). The study sites were chosen to encompass a range of primary malaria vector distribution, taking into account rates of malaria incidence, previous results regarding pyrethroids and DDT resistance and biochemical mechanisms, easy access by land or water, safety, and public health priority in terms of resistance monitoring given the history of insecticide use [21]. The main insecticides used in these areas for malaria control are deltamethrin, lambda-cyhalothrin, alphacypermethrin, permethrin and fenitrothion.

**Table 1:** Number of individuals sequenced by *Anopheles* species by locality with their corresponding access numbers to GenBank

*Anopheles* specie	Locality, Municipality, Department	Number of sequenced specimens		GenBank Access number
		Resistant phenotype	Susceptible phenotype	
*An. darlingi*	Bocas de Puné, Medio Atrato, Chocó	9	11	MN0503065, MN0503066, MN0503069, MN057656, MN062206, MN062226–MN062240
	Encharcazón, Río Iró, Chocó	4	4	MN057652, MN057660, MN062210, MN062219, MN062222–MN062225
	Tagachí, Quibdó, Chocó	28	27	MN053063, MN053064, MN053067, MN053068, MN057644–MN057651, MN057653–MN057658, MN057661, MN057662, MN062207–MN062209, MN062211–MN062218, MN062220, MN062221, MN062241–MN062262
***Subtotal***		***41***	***42***	
*An. nuneztovari* s.l.	Córdoba, Buenaventura, Valle de Cauca	10	11	MN076484–MN076486, MN076489–MN076493, MN076498, MN087492, MN087494–MN087503
	Santa Rosa, El Zulia, Norte de Santander	2	3	MN076487, MN076494–MN076497
***Subtotal***		***12***	***14***	
*An. albimanus*	Panguí, Nuquí, Chocó	9	11	MN087515–MN087520, MN087504–MN087510, MN087523
***Subtotal***		***9***	***11***	
***Total***		***62***	***67***	

The mosquitoes of these locations were previously characterized as resistant populations as follows: *An. albimanus* from Panguí (Nuqui-Chocó) was resistant to lambda-cyhalothrin and DDT with a range of 92% and 98% mortality in the bioassays; *An. darlingi* from Bocas de Pune (Medio Atrato—Chocó), Tagachi (Quibdó-Chocó) and Encharcazón (Rio Iró-Chocó) were resistant to deltamethrin, lambda-cyhalothrin, permethrin and DDT with mortalities ranging between 80 and 97%, and *An. nuneztovari* from Santa Rosa (El Zulia-Norte de Santander) and Córdoba (Buenaventura-Valle del Cauca) were resistant to deltamethrin and DDT, with mortalities ranging between 94 and 98% [21]. When quantifying the intensity of these resistance, all of these populations were found with low resistance intensity to the pyrethroid insecticides (alpha-cypermethrin, deltamethrin, lambdacyhalothrin, permethrin) and the organochlorine DDT. A detailed description of the sampling sites and the results of the biological tests are provided in a previous paper [21]. Although identifying the presence of *kdr* mutations in *An. albitarsis* s.l. was not the subject of this study, mosquitoes belonging to these species from the Santa Rosa locality that survived to diagnostic concentrations in the biological tests were sequenced (MN108498–MN108503).

### Molecular assays to identify mutations of the VGSC gene

To identify the association between resistant phenotypes and *kdr*-type genotypes, surviving (n = 62) and dead (n = 67) *An. nuneztovari* s.l., *An. darlingi* and *An. albimanus* mosquitoes were used to identify point mutations at the S6 segment of domain II of the VGSC gene at codons 1010, 1013 and 1014 which have been associated with insecticide resistance in different species of *Anopheles* malaria vectors.

#### Genomic DNA extraction

Genomic DNA extractions from individual mosquito samples were performed using the Qiagen’s DNeasy^®^ Blood & Tissue Kit (Qiagen, Hilden, Germany) following the manufacturer’s instructions with some modifications related with centrifugation times and the elution volume. The time was reduced in all cases by half and the final volume of eluted was 150 µl. The amount of DNA was variable between samples. The ratio of absorbance at A260/A230 and A260/A280 nm was used to assess the concentration and purity of each DNA sample using a NanoDrop 2000 spectrophotometer (Thermo Fisher Scientific).

#### PCR amplification of segment 6 of domain II of the VGSC gene

A ~ 225 bp fragment of the kdr region in the VGSC gene, spanning codons 1010, 1013 and 1014 of *An. darlingi* (228 bp, between exons 20 and 21)*, An. albimanus* (225 bp, between exons 22 and 23) and *An. nuneztovari* s.l. (226 bp), was amplified using primers designed for *An. albimanus*, AAKDRF2 (5′CATTCATTTATGATTGTGTTTCGTG3′) and AAKDRR (5′GCAANGCTAAGAANAGRTTNAG) [18]. The PCR mixture (50 µl) consisted of 0.5–2.0 µmol/ml DNA template, 1 U/µl GoTaq^®^ G2 Flexi DNA Polymerase (Promega), 1× Green GoTaq^®^ Flexi Buffer, 0.5 mM dNTPs, 2.5 mM MgCl_2_, 1.5 μM AAKDRF2 and 1.5 µM AAKDRR. The reaction program was 95 °C for 3 min, followed by 35 cycles each with 95 °C for 1 min, 45 °C for 1 min, 72 °C for 1 min, and by a final extension of 10 min at 72 °C. PCR products were analysed by electrophoresis in 2.5% (w/v) agarose gel containing SafeView Plus (Fermelo Biotec).

#### Purification of DNA fragments from PCR

DNA fragments from PCR were purified using the QIAquick PCR Purification Kit (Qiagen, Hilden, Germany). Nucleic acid concentrations of purified samples were measured using a Synergy 2 microplate reader (Biotek). The final concentration of 50 ng/µl for each of the samples was adjusted according to the requirement of the sequencing service. Some samples were sent to be sequenced having a minimum concentration of 20 ng/µl.

#### Sequencing PCR products

PCR products were sequenced using the forward AAKDRF2 and reverse primer AAKDRR. Some of the sequencing reactions were performed at Macrogen Inc (South Korea) (n = 50) and others were performed at the Roy J. Carver Biotechnology Center at the University of Illinois (n = 79). Contig and peak chromatogram verification were done using the SeqMan module of the LaserGene v8.1 suite (DNASTAR Inc. Madison, WI, USA). Polymorphisms at codons 1010, 1013 and 1014 which have been implicated in insecticide resistance in several *Anopheles* species were checked manually. The identity of the sequenced fragments was checked by comparison with the existing VGSC gene sequences of *Anopheles* species at the GenBank using the NCBI Blastn tool. All generated sequences were aligned using Clustal W in MEGA, version 7.0.26 [23]. DNA sequences were deposited in GenBank.

## Results

**Fig. 1:**
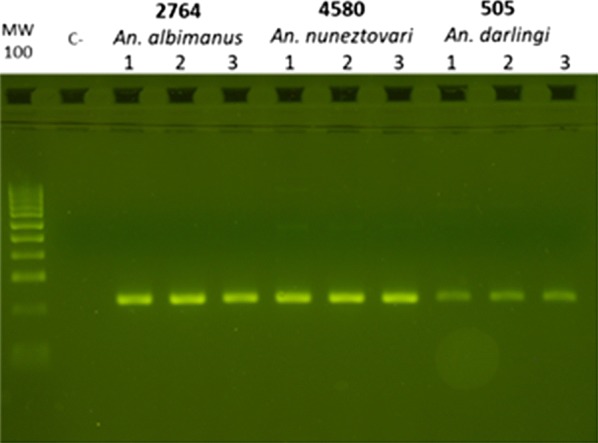
Photograph of an agarose gel electrophoresis showing the PCR products amplified (approx. 225 bp) with AAKDRF2 and AAKDRR PCR primers. In lines 3, 4 and 5 *An. albimanus*; In lines 6, 7 and 8 *An. nuneztovari* s.l. and lines 9, 10 and 11 *An. darlingi*. In line 1 the molecular-weight size marker appears every 100 base pairs and in lane 2 the negative control

A total of 129 mosquitoes, 83 *An. darlingi*, 26 *An. nuneztovari* s.l., and 20 *An. albimanus* were amplified and genotyped at the kdr region with this approach (Fig. 1 and Table 1). All samples were sequenced in the forward and reverse direction; however, as some chromatograms had noisy sequence peaks with low quality scores, 92 samples have consensus sequences assembled from forward and reverse sequences, 22 were sequenced with the forward primer and 15 with the reverse primer. Forward and reverse Sanger sequencing allowed identification of the codons of interest. The sequences of all 129 samples were aligned along with sequences of *Anopheles sinensis* (KP763768), *Anopheles subpictus* (KF023519), *Anopheles punctipennis* (AY283041), *An. albimanus* (KF137581), *An. darlingi* (JQ658981–JQ658985) and *Anopheles marajoara* (JQ658986–JQ658989) available in GenBank in order to identify intron/exon borders.

Regarding the three codons, in which mutations associated with resistance to pyrethroid insecticides and DDT have been identified in other malaria vectors, it was evidenced that only the GTT codon for valine at position 1010 (V1010), the AAC codon for asparagine at position 1013 (N1013) and the TTA and TTG codons, both of which code for leucine at position 1014 were present; indicating that no amino acid mutation was detected in the IIS6 sequences of any mosquitoes processed in this study. At the position 1014, the TTA codon was the only one detected in *An. nuneztovari* s.l. and *An. darlingi* and only the TTG codon in *An. albimanus*.

**Fig. 2:**
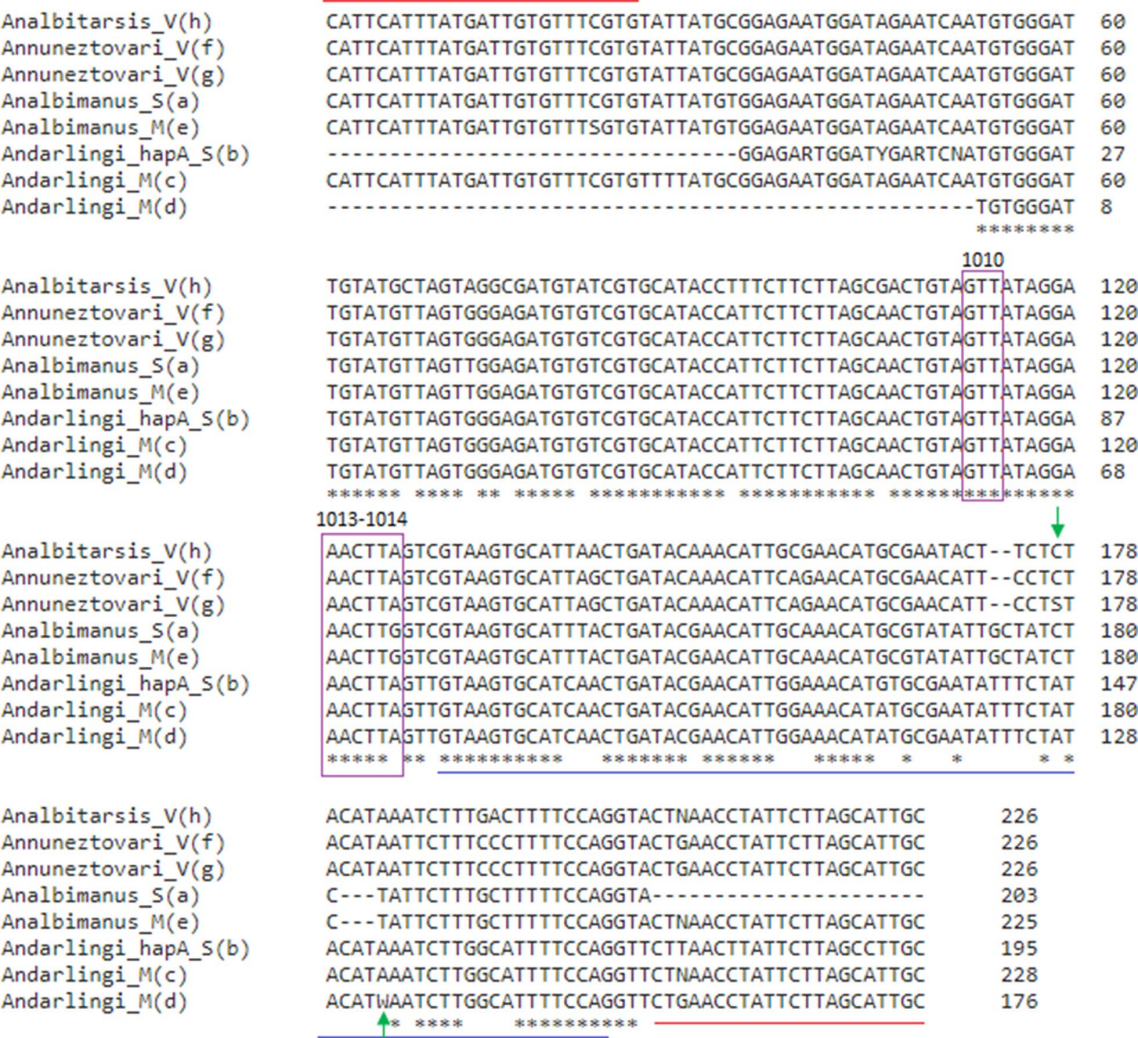
Alignment of the sequences of *An. albimanus* (e) (MN087505), *An. darlingi* (c, d) (MN053065, MN062219), *An. nuneztovari* s.l. (f, g) (MN076484, MN076491) and *An. albitarsis* s.l. (h) (MN108499) obtained in this study, with sequences of *An. albimanus* (a) (KF137581.1) and *An. darlingi* (b) (JQ658981.1) available at the GenBank. The identical positions are indicated by an asterisk and mutation sites reported for other *Anopheles* species are enclosed by a box. A blue line below the sequence indicates intron position. Primers AAKDRF2 (5′-CATTCATTTATGATTGTGTTTCGTG-′3); AAKDRR (5′-GCAANGCTAAGAANAGRTTNAG-′3) used to amplify the segment are indicated by red arrows. SNPs detected in the intron are indicated with a green arrow

**Fig. 3:**
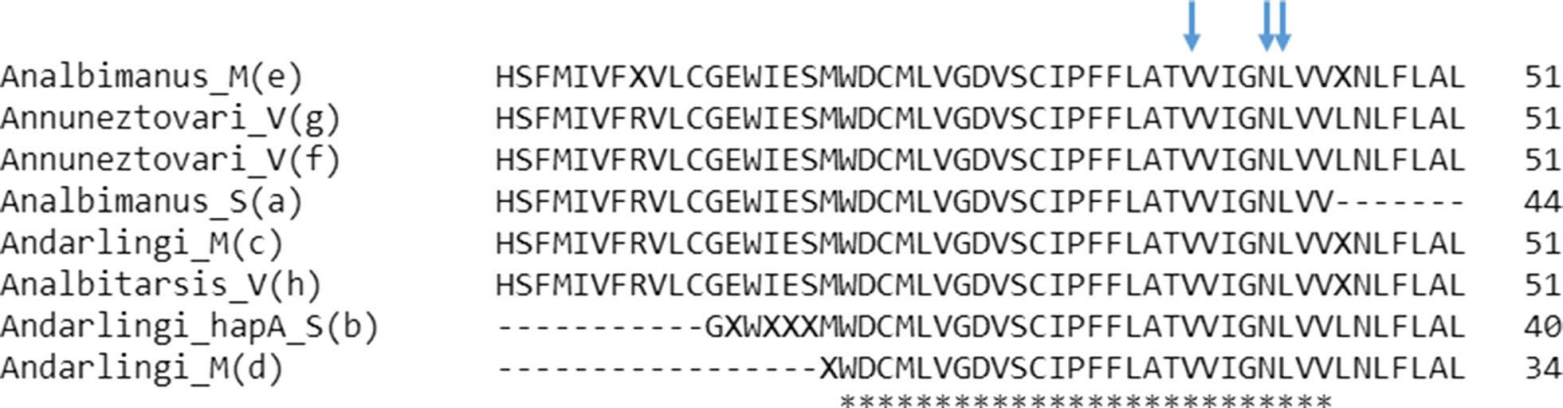
*Anopheles albimanus* (MN087505), *An. darlingi* (MN053065, MN062219), *An. nuneztovari* s.l. (MN076484, MN076491) and *An. albitarsis* s.l. (MN108499) VGSC protein sequence alignment. Blue arrows indicated the position of the amino acid where mutations have been detected

Sequence analyses of intron, located just downstream of the *kdr* mutation site, showed that it varied in sequence and size between species (Fig. 2). In *An. darlingi*, the intron (74 bp) is larger than in *An. nuneztovari* s.l. (72 bp) and *An. albimanus* (71 bp) (Fig. 2). Few intra-specific differences were observed in the intron of the sequenced samples. One single nucleotide polymorphism (SNP) was present in one sample of *An. darlingi* with susceptible phenotype from Bocas de Pune: heterozygous for A/T at position 56. Another SNP was also present in *An. nuneztovari* s.l. individuals at position 50 where it was evidenced a heterozygous for G/C in most of the samples regardless of the locality of origin and the phenotype evidenced in the biological tests of susceptibility. In general, the analyses of the partial sequence of exons showed that even though there are variations in the nucleotide sequences of the three species, these variations did not produce changes in the amino acid sequence (Fig. 3). In *An. albitarsis* s.l. from the locality of Santa Rosa no mutations were observed and only one haplotype was present.

## Discussion

The molecular analysis of the a ~ 225 bp fragment that encodes for segment IIS6 of the VGSC gene indicated that the 1010L, 1013S, and 1014F/S/C/W polymorphisms reported in other *Anopheles* species are not found in pyrethroid and DDT resistant populations of *An. albimanus*, *An. darlingi* and *An. nuneztovari* s.l. from malaria-endemic areas of Colombia [21]. This suggest that metabolic resistance mechanism reported previously in these species might be responsible for the resistance observed [11, 12].

In *Anopheles* species, nine naturally occurring mutations in the sodium channel in the positions V1010 (L), N1013 (S), L1014 (F/S/C/W), I1048 (N), S1156 (G), and N1575 (Y) have been described and associated with resistance phenotype. Among them, only the mutations in the positions 1014 have been expressed in *Xenopus* oocytes, confirming their role in the reduction of the pyrethroid sensitivity of the sodium channel [15]. All the mutations mentioned have been reported in malaria vectors from Asia and Africa, with only L1014F and L1014C reported for *An. albimanus*, a key malaria vector in Latin America [18]. There are other mutations along the sodium channel that have been examined in *Xenopus* oocytes and have been associated with knockdown (*kdr*) resistance to pyrethroids in various arthropod species including disease vectors [15]. As only a small region of the VGSC gene was evaluated in this study, it could not be ruled out the possibility that the observed simultaneous resistance to pyrethroids and DDT in some populations was associated with mutations present in a different region of the gene. In order to explore this, a longer region of the gene should be amplified.

Metabolic mechanisms were found in resistant populations of *An. darlingi* and *An. nuneztovari* s.l. in Colombia. Increased levels of mixed function oxidases were found in the resistant population of *An. darlingi* in Choco, where cross-resistance between lambdacyhalotrin and DDT was observed [12]. For pyrethroid resistant populations of *An. nuneztovari* s.l., increased levels of non-specific esterases was found [11]. These previous findings point out the presence and importance of metabolic mechanisms in resistant populations of *An. darlingi* and *An. nuneztovari* s.l. in Colombia. Studies carried out in other regions and with different *Anopheles* species have shown the importance of metabolic detoxification of pyrethroids, especially mixed function oxidases [24–31], and also, the presence of other resistance mechanisms, such as reduced penetration [32–34], which confer high levels of resistance, even in the absence of *kdr* mutations [30].

Relatively few data about resistance mechanisms are available for the most important Latin American malaria vectors. In fact, with the exception of the tests carried out with *An. darlingi* and *An. nuneztovari* s.l. in Colombia, all studies have been done with *An. albimanus* and most of them were made more than 10 years ago. Metabolic resistance is the most studied mechanism. *An. albimanus* populations from Guatemala, Panamá, El Salvador and Mexico demonstrated elevated levels of AChE responsible for the resistance to organophosphates and carbamates [4, 35–38] and populations from Mexico evidenced increased activity of Glutathione *S*-transferase, Cytochrome P450 and Esterase in DDT and pyrethroid resistance [4, 38]. Regarding target site resistance, mutations in *ace*-*1* gene (G119S and G119A) were consistently associated with resistance to organophosphates and carbamates in *An. albimanus* populations from Peru [39] and mutations in VGSC gene (L1014F and L1014C) were related with pyrethroid resistance in Mexico, Costa Rica and Nicaragua [18]. More recent studies using whole transcriptome sequencing have shown that the cytochrome P450 CYP9K1 was overexpressed in deltamethrin and alpha-cypermethrin-resistant samples from Peru and in deltamethrin-resistant samples from Guatemala and CYP6P5 overexpression in deltamethrin-resistant samples from Peru. In that same study, *kdr* mutations were also detected at the L1014S/C position in deltamethrin and alpha-cypermethrin-resistant samples from Peru [40]. Additionally, studies of the microbiota in mosquitoes of differing insecticide resistance status have showed differing composition of the microbiota and its functions between fenitrothion-susceptible and fenitrothion-resistance mosquitoes from Peru [41].

It is possible that studies on insecticide resistance mechanism have been limited in the Americas region due to the high diversity of species, low mosquito density and the requirements for the maintenance and transport of samples. Particularly in Colombia, the areas of highest malaria transmission, which have been continuously treated with insecticides, have scattered settlements, difficult access conditions and frequent public order problems or illegal mining. This have made it difficult to perform tests to detect insecticide resistance mechanisms in a regular basis.

In Colombia, this is the first study aimed to associate the resistance to pyrethroid insecticides and DDT, detected in the main malaria vectors, with the mutations in the VGSC gene most frequently reported in species of the genus *Anopheles.* Although, there was no evidence of these mutations in the evaluated populations, this is the first report of this fragment in *An. nuneztovari* s.l., an important regional malaria vector. This study demonstrated that the primers previously reported [18] can be used for the amplification of this fragment in four important vectors of malaria in the Americas—*An. albimanus*, *An. darlingi*, *An. nuneztovari* s.l., and *An. albitarsis* s.l.—with some modifications in the reverse primer to reduce the number of degenerate bases (AAKDRR2: 5′-GCAATGCTAAGAATAGGTTNAG-′3) and optimize primer annealing.

The results of this study should be interpreted with caution for two reasons: first, the purpose was to relate *kdr* genotypes with their phenotypic outcome previously observed [21]; so the *Anopheles* samples analysed from each locality were relatively few, and it is important to expand the analyses in order to include more samples. Second, because the amplified fragment was short (~ 225 bp), it is not possible to dismiss the possibility that other mutations along the VGSC gene may be related to the DDT and pyrethroid resistance observed in these vectors; as it has been reported in other species of the genus *Anopheles* or in other insect vectors of human diseases [15].

Additionally, accordingly Lol et al. [18] and Henry-Halldin et al. [42], this study provide evidence about the inter-specific differences in the intron size and nucleotide sequence positions and support its utility for taxonomic classification. However, it is unknown if those variations allow discrimination to species complexes level such as *An. nuneztovari* s.l. and *An. albitarsis* s.l.

Measuring the intensity of resistance in malaria vectors was recently included in the WHO procedures [43] as an additional bioassay to measure whether or not resistant mosquitoes could survive to higher insecticide concentrations. The interpretation of these observations are linked to possible failure in the field, and indeed, good correlation has been observed between high intensity resistant populations and failure of programmatic applications of insecticides for malaria control [44]. In the study presented here, although the resistance intensity was low, all populations were resistant to pyrethroids [21], and in the absence of *kdr* mutations, previously reported metabolic mechanisms [11, 12] are likely to be responsible for the resistance observed.

## Conclusion

Mutations frequently associated with knockdown (*kdr*) resistance to DDT and pyrethroid insecticide were absent in the resistant populations of the main malaria vectors, *An. darlingi*, *An. nuneztovari* s.l., and *An. albimanus* in Colombia. These results suggest that previously reported metabolic resistance mechanisms might be responsible for the resistance observed in these populations. Although, even more studies are required to identify the underlying mechanisms that are behind the resistance to the insecticides detected in the present work, this is the first study that aims to determine whether the *kdr* mutations are also associated with resistance to DTT and pyrethroids insecticides in the main malaria vectors of Colombia. It is also important to mention, that the knowledge of the mechanisms involved in the resistance to insecticides, as it is presented in the study, can provide valuable information to improve the strategies of the management of insecticides in order to become more effective the malaria vector control program.

## Data Availability

All data generated or analyzed during this study are included in this published article.

## Abbreviations

DDT: dichlorodiphenyltrichloroethane (IUPAC name: 1-chloro-4-[2,2,2-trichloro-1-(4-chlorophenyl)ethyl]benzene)
DNA: deoxyribonucleic acid
Kdr: knockdown resistance
MACE: modified acetylcholinesterase
MFO: mixed function oxidases
NCBI: National Center for Biotechnology Information
NSE: non-specific esterases
SNP: single nucleotide polymorphism
VGSC: voltage-gated sodium channel
WHO: World Health Organization
